# Differential pathogenicity and lethality of bubonic plague (1720–1945) by sex, age and place

**DOI:** 10.1098/rspb.2024.0724

**Published:** 2024-07-24

**Authors:** J. Mongillo, N. Zedda, N. Rinaldo, T. Bellini, M. C. Manfrinato, Z. Du, R. Yang, N. C. Stenseth, B. Bramanti

**Affiliations:** ^1^ Department of Environmental and Prevention Sciences, University of Ferrara, Ferrara 44121, Italy; ^2^ Department of Neurosciences and Rehabilitation, University of Ferrara, Ferrara 44121, Italy; ^3^ University Strategic Center for Studies on Gender Medicine, University of Ferrara, Ferrara 44121, Italy; ^4^ State Key Laboratory of Pathogen and Biosecurity, Beijing Institute of Microbiology and Epidemiology, Beijing, People‘s Republic of China; ^5^ Center for Pandemics and One Health Research, Sustainable Health Unit (SUSTAINIT), Faculty of Medicine, University of Oslo, Oslo 0316, Norway; ^6^ Centre for Ecological and Evolutionary Synthesis, Department of Biosciences, Faculty of Mathematics and Natural Sciences, University of Oslo, Oslo 0316, Norway; ^7^ Vanke School of Public Health, Tsinghua University, Beijing 100084, People‘s Republic of China

**Keywords:** *Yersinia pestis*, epidemiology, sex-disaggregating selection

## Abstract

COVID-19 brought back to the attention of the scientific community that males are more susceptible to infectious diseases. What is clear for other infections—that sex and gender differences influence both risk of infection and mortality—is not yet fully elucidated for plague, particularly bubonic plague, although this knowledge can help find specific defences against a disease for which a vaccine is not yet available. To address this question, we analysed data on plague from hospitals in different parts of the world since the early eighteenth century, which provide demographic information on individual patients, diagnosis and course of the disease in the pre-antibiotic era. Assuming that the two sexes were equally represented, we observe a worldwide prevalence of male cases hospitalized at any age, a result which seems better explained by gender-biased (thus cultural) behaviours than biological sex-related factors. Conversely, case fatality rates differ among countries and geographic macro-areas, while globally, lethality appears slightly prevalent in young females and older adults (regardless of sex). Logistic regression models confirm that the main risk factor for bubonic plague death was the geographical location of the cases and being older than 50 years, whereas sex only showcased a slight trend.

## 1. Introduction

Molecular evidence has shown that *Yersinia pestis* has affected people in Eurasia since the Neolithic and through the Bronze Age [1–5]. Conventionally, we distinguish three major plague events, called pandemics since they spread over two or more continents, although not continuously: the First Plague Pandemic (sixth and seventh centuries) hit Africa and Eurasia, the Second Plague Pandemic (1346−nineteenth century) spread from Central Asia [6,7] throughout Europe and to sub-Saharan Africa [8], whereas the Third Plague Pandemic, which has its origin in 1772 in the Yunnan region of China, spread all over the world from Hong Kong in 1894 [6,9]. Several ancient DNA studies have demonstrated that *Y. pestis* was the causative agent of all historic pandemics [10–25]. The ability of *Y. pestis* to generate three distinct forms of plague (bubonic, septicaemic and pneumonic) was also genetically acquired during its evolution time (reviewed in [26]). The bacillus adapted to distinct mechanisms of transmission mediated by parasites’ bites (bubonic when intradermic inoculation occurs, and septicaemic if *Y. pestis* enters the bloodstream), or by respiratory droplets (pneumonic plague) (e.g. [6,27]). In the bubonic form of plague, once the bacterium is inoculated intradermally, it should be cleared in the skin by the cells of the innate immune system recruited at the site of the wound [28,29]. From murine models, we know that neutrophils are the first arriving at site within few minutes or hours [30] and are important in this early phase and for the first 1–2 days after inoculation to control the development of bubonic plague possibly by apoptosis [29], whereas the transport of *Y. pestis* seems to depend on macrophages also recruited at site [30,31]. Using isolated human polymorphonuclear leukocytes incubated at 21°C, Spinner *et al*. [32] demonstrated that apoptotic neutrophils containing survived *Y. pestis* can be internalized by macrophages, in which they remain viable and replicate. In fact, *Y. pestis* is able to neutralize the phagocytosis process in the host’s macrophages (e.g. [27,33–36]) and the transit in the flea’s gut seems to enhance *Y. pestis* resistance to phagocytosis by macrophages [37]. Within the first 6–12 h, dying macrophages convey and release the bacteria to the next draining lymph node [30], where the pathogens start multiplying extracellularly, after having neutralized the phagocytotic activity of the neutrophils [38,39]. Bacteria multiplying in the lymph node cause the formation of a swollen, painful bubo. The necrosis of the lymph node causes bacteraemia, septicaemia and the production of endotoxins quickly producing shock, disseminated intravascular coagulation, coma and death [40]. Without treatment with antibiotics (introduced no earlier than the late 1940s [41]), the three forms differ in their mortality rate as well, being close to 100% for septicaemic and pneumonic plague and about 40–70% for bubonic plague [6].

Previous studies have tried to address in different populations questions about biological (versus cultural) parameters that might have driven patterns of plague lethality. In particular, sex, age, condition of frailty, as well as environmental factors (reviewed in [42], but also see [43]) have been considered potential biological causes of selective lethality. Sex-related mortality owing to plague has been investigated extensively using bio-archaeological markers on skeletons of plague victims, as well as mortality records (reviewed in [42], but also see [44–49]). All these works, which have reached controversial and non-univocal conclusions, were carried out on singular or multiple archaeo-anthropological and historical datasets. Some of these works have evidenced in early modern records a sex-disaggregating selection against females [50–55], whereas no trend was evidenced from the biological profile of plague victims of different epochs considered together [42]. The only trend discovered in this last complex dataset concerned a prevalence of deaths among individuals 5–10, but mostly 20–35 years of age [42]. The absence of any other detectable biological trend among different plague cemeteries of different epochs let the authors conclude that the differences in sex ratio observed in single graveyards may have been owing to varying compositions of the populations, a hypothesis which is also supported by recent historical work [56,57]. Another possibility is that distinct cultural behaviours of the two genders, with particular reference to their occupational activity (e.g. [57,58]) or gender-based discrimination or inequities (e.g. [55]) may have enhanced the risk of exposition and/or the risk of death for one gender or the other in the population under investigation. Nonetheless, some limitations are intrinsic to studies carried out on skeletal remains of plague victims and might have constrained the informativeness of the dataset itself [42]. In particular, the assumption of causality linking the number of infected and the risk of death, owing to the impossibility of calculating the case fatality rate (CFR, expressed by the percentage of deaths among cases). Moreover, the actual form of plague (bubonic, septicaemic or pneumonic) that caused the death of victims can only be conjectured from archaeological skeletons, as well as from many historical data of the pre-industrial era.

To overcome this constraint, we collected historical clinical data on plague patients from the period 1720 to 1945 from different parts of the world. Compared with earlier data, these modern records contain accurate information about sex and age of individual plague cases, diagnosis of the plague form the patients were suffering from, and their outcome in terms of death or recovery after infection. This dataset enabled us to calculate the CFR of bubonic plague for the two sexes, at different ages and in different countries. Further, having data from different geographical areas and different periods, we could discuss whether biological factors (resulting in homogeneous trends) or cultural factors (resulting in non-homogeneous trends) may have influenced our observations on pathogenicity and lethality of plague. From the analysis of the dataset, we observed that among the hospitalizations, males infected by plague, in particular bubonic plague, were prevalent, a situation that may possibly be more influenced by gender-related cultural reasons—whereas the slightly higher CFR observed in females might tentatively be better explained by biological factors. Local cultural differences may have influenced the great dissimilarities observed in mortality among geographic macro-areas—albeit genetic reasons could not be completely excluded by this study.

## 2. Results

**Figure 1.**
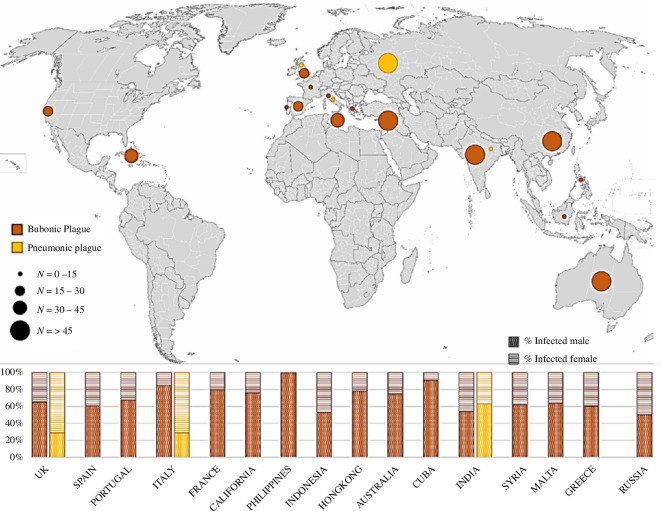
Spatial distribution and incidence of pneumonic and bubonic clinical plague cases collected from the literature for this study (Second and Third Pandemics). At the bottom of the panel are resumed percentages of cases disaggregated by sex for each country.

The complete dataset (electronic supplementary material, table S1) contains 1616 cases recorded during the eighteenth−twentieth centuries in hospitals of 17 countries on 5 continents. Of the 1086 male and 530 female cases, the only 100 who were affected and died of septicaemic plague were scattered in different geographical areas, thus not relevant for our purposes. Moreover, as the lethality is very close to 100% [59,60], septicaemic plague cases do not help to distinguish CFRs by sex and age (electronic supplementary material, S1). Septicaemic cases were therefore not considered for further analyses. The spatial distribution of the remaining bubonic and pneumonic cases is given in figure 1, while the relative data are summarized in electronic supplementary material, table S2.

We further excluded 362 cases of pneumonic plague from the subsequent analyses on plague lethality, because the probability of dying from pulmonary plague was close to 100% in all countries as well—irrespective of sex and age. The only exception we found (electronic supplementary material, tables S2 and S3) was India during the Third Pandemic, but the number of cases recorded was too small (eight cases and five deaths) for the CFR to be considered representative. In our dataset, pneumonic plague cases were in general underrepresented during the Third Pandemic in comparison with Europe during the Second Pandemic (mainly represented by the outbreak of Vetlianka 1878–1879 [61]).

Consequently, we limited subsequent analyses to only those bubonic plague cases whose diagnosis had been explicitly stated in hospitals’ reports. Based on where the bubonic plague cases were reported, we also divided the data into four geographical macro-areas: Europe and the Mediterranean (which included data from the United Kingdom, Spain, Portugal, Italy, France, Malta and Greece, as well as Syria [61–78]); India [79]; East Asia (which included data from Philippines, Indonesia and Hong Kong [73,80–82]); and Australia [83–85] (table 1). Data from California and Cuba [86–88] (54 cases) were excluded from the subsequent dataset because they were insufficient to represent that of the United States.

**Table 1.** Percentual CFR of bubonic plague cases of the Second and Third Pandemics in different geographic macro-areas, subdivided by sex. The prevalent values are indicated in bold.

	cases		deaths		CFR%			***p*-value^a^**
	males	females	males	females	males	females	M&F	
Europe II P	132	69	73	42	55.3	**60.9**	57.2	0.694
East Asia III P	171	50	131	37	76.6	74.0	76.0	0.885
Europe III P	91	26	36	15	39.6	**57.7**	43.6	0.319
India III P	117	99	66	68	56.4	**68.7**	62.0	0.371
Australia III P	259	86	88	22	34.0	25.6	31.9	0.290
total	770	330	352	166	45.7	**50.3**	47.1	0.439
%	**70%**	30%	51.2%	**55.6%**				

^a^ Comparison between males and females (chi-squared test).

In table 1, we summarize data and CFRs of all clinical cases of bubonic plague. The global CFR in the period considered is 47.1%, demonstrating that almost half of the hospitalized people affected by bubonic plague died worldwide. However, for the Third Pandemic (i.e. after 1894), the CFRs in the four macro-areas differ considerably, ranging from 31.9% in Australia, 43.6% in Europe, 62.0% in India, up to 76.0% in East Asia. This result showcases that mortality in Australia was extremely low compared with East Asia during the Third Pandemic. In Europe, we observed a reduction in mortality in the Third Pandemic in comparison to the Second Pandemic (43.6% versus 57.2%, respectively), yet the difference is not statistically significant (*p* = 0.183).

With regard to the data disaggregated by sex, we also observed that the absolute frequencies in all macro-areas showed a prevalence of male cases (770 infected males against 330 infected females were hospitalized, corresponding to 70% males against 30% females). The difference is relevant, especially when assuming a balanced sex ratio in the populations. This confirms the data observed in the histograms in the lower part of figure 1, where we see that the frequency of male cases is always higher than that of females in almost all countries, with the exception of Russia, for which the number of males and females is equivalent. In table 1, we noted that the CFRs differed substantially across the four macro-areas during the Third Pandemic, with a prevalence of female deaths in Europe (57.7% versus 39.6%) and India (68.7% versus 56.4%), and a prevalence of male deaths in Australia (34.0% versus 25.6%) and East Asia (76.6% versus 74.0%). However, the differences between the sexes are not statistically significant in any macro-area as well as in the global sample (table 1). Comparing the CFRs of the two sexes separately in Europe for the two pandemics, we also found no statistical difference (males: *p* = 0.211; females: *p* = 0.962). Therefore, we decided to merge the data from the two pandemics in Europe for the subsequent analyses.

Globally, we observed that females tended to die more from bubonic plague than males, albeit the difference is not statistically significant (table 1).

From this reduced dataset of 1100 cases of bubonic plague, with known sex, we further selected only those patients whose individual age was also known (967 individuals). Doing so, the total CFR ratio does not change (50.4% in males and 54.7% in females; table 2) and we were able to disaggregate the dataset into 10-year age groups, to observe whether there was a trend in their respective CFRs (table 2). Data not disaggregated by sex showcase slight differences in lethality among age classes, but for the older adults (50+), who have the highest CFR (61.4%), and for the 10–19 age class, who showed the lowest value of CFR (45.2%). Interestingly, while the CFR of the older adults slightly differs between the sexes (61.7% in males versus 60.9% in females), those of the subadults diverge by sex, albeit not significantly, with prevalent CFRs in females in both classes 0–9 years and 10–19 years.

**Table 2.** Subdivision per sex and 10-year age classes of all cases of bubonic plague (Second and Third Pandemics merged). The prevalent CFRs are indicated in bold.

	males			females			total sample			*p*-value^a^
	cases	deaths	CFR%	cases	deaths	CFR%	cases	deaths	CFR%	
total	667	336	50.4	300	164	**54.7**	967	500	51.7	0.560
50+	47	29	**61.7**	23	14	60.9	70	43	61.4	0.862
40–49	70	36	**51.4**	45	23	51.1	115	59	51.3	0.885
30–39	118	59	50.0	42	22	**52.4**	160	81	50.6	0.997
20–29	205	115	**56.1**	81	43	53.1	286	158	55.2	0.890
10–19	185	76	41.1	78	43	**55.1**	263	119	45.2	0.254
0–9	42	21	50.0	31	19	**61.3**	73	40	54.8	0.751

^a^ Comparison between males and females (chi-squared test).

**Figure 2.**
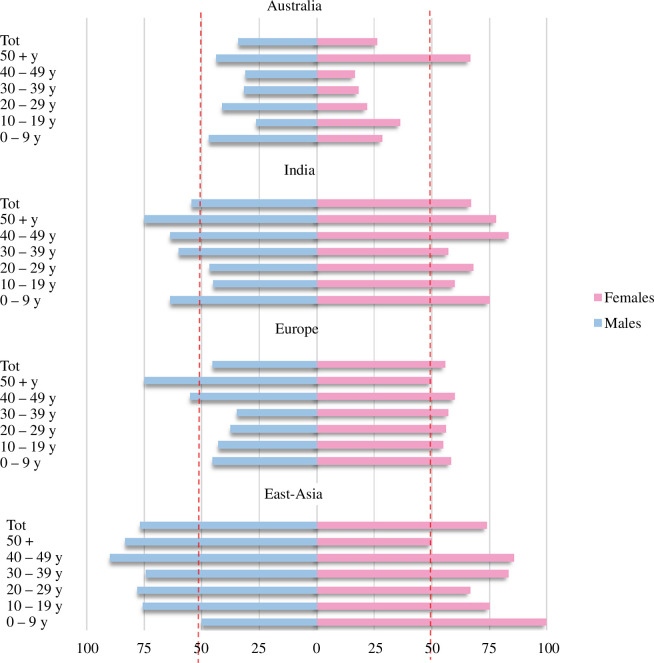
CFRs subdivided for different geographic macro-areas, and by age (10-year groups) and sex (in blue, males; in pink, females) for bubonic plague. Dashed red lines mark the 50% of CFR for females and for males. Data for Europe from the Second and Third Pandemics were merged.

Furthermore, we drew histograms of the CFRs clustered in the 10-year groups for each macro-area (figure 2). The CFR value in Australia and East Asia showed a bias towards males (with the only exception of the 50+ group in Australia and children 0–9 years in East Asia), whereas in India and Europe, the bias is towards females (only exception, the class 50+ in Europe).

**Figure 3.**
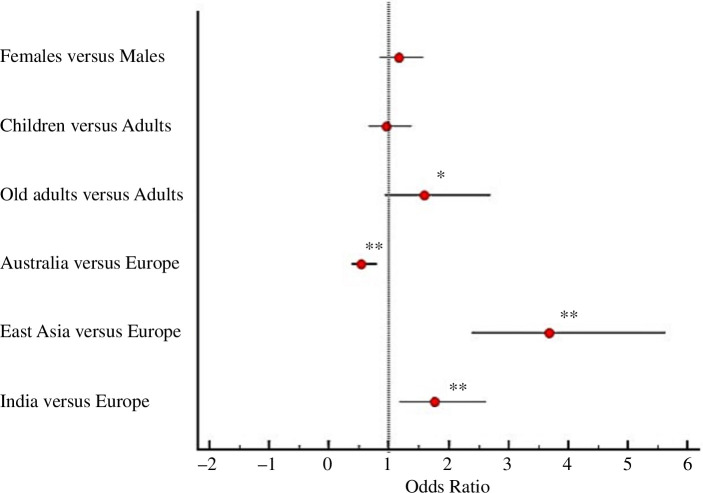
Forest plot displaying the OR of being dead of plague versus being recovered from plague. An OR greater than 1 indicates a higher probability of dying of plague, while an OR less than 1 indicates a lower probability of dying of plague and a higher probability of recovering. Red dots indicate OR values. Horizontal bars represent 95% CI. *p < 0.1; **p < 0.01 (table 3).

To enhance our understanding of bubonic plague lethality, we estimated the odds ratio (OR) of dying by bubonic plague using a logit model and adding as independent variables sex, age classes and geographic macro-areas. We decided to use as an independent variable the biological age, intended as childhood (0–14 years), adulthood (15–49 years) and ageing (50+), to test the influence of biological and physical changes owing to hormonal fluctuations (electronic supplementary material, table S3). As reported by the logit model (table 3) and the forest plot (figure 3), even if females appear to have slightly higher odds of dying of plague than males, the odds are not significantly different between the two sexes; on the other hand, older adults had significantly higher odds of dying in respect to adults (more than 1.5 times the odds). Moreover, significant ORs are reported for the four geographic macro-areas (table 3). Getting sick in East Asia and India increases the risk of dying compared with Europe (used as a reference), while being affected in Australia decreases the risk of dying. The overall variance explained by the model is 16% (based on Nagelkerke *R*^2^). As reported by the Hosmer and Lemeshow test (*p* > 0.05), the fitted model is adequate. Further attempts considering data only from the Third Pandemic or from the Second and Third Pandemics separately in Europe did not show relevant changes (electronic supplementary material, tables S4 and S5).

**Table 3.** Logistic regression model (logit) representing the relative OR of dying by plague in relation to individuals’ biological characteristics and geographical area. The 95% confidence intervals (95% CI) are reported in parentheses.

variables	OR (95% CI)	*p*‐value
**sex**		
males	1 (reference)	—
females	1.156 (0.861; 1.553)	0.335
**biological age classes**		
children (0−14 years)	0.950 (0.670, 1.349)	0.775
adults (15−49 years)	1 (reference)	—
old adults (50+ years)	1.583 (0.934; 2.683)*	0.088
**geographic area**		
Europe (II+III pandemic)	1 (reference)	—
Australia	0.541 (0.376; 0.779)***	0.001
East Asia	3.675 (2.401; 5.625)***	<0.001
India	1.756 (1.183; 2.609)***	0.005
*R*^2^ Cox and Snell	0.12	
*R*^2^ Nagelkerke	0.16	
**Hosmer and Lemeshow test**		
chi-squared	8.688	
d.f.	7	
*p*‐value	0.276	

*p < 0.1; **p < 0.05; ***p < 0.01.

## 3. Discussion

On the occasion of the COVID-19 pandemic, a strong bias towards older people and males was observed worldwide [89], recalling the general rule that males are more susceptible to be infected by pathogens and to have more negative outcomes than females (e.g. [90–95]). Although epidemic-prone infectious diseases like TBC, malaria and leishmaniosis have been proven to follow this general rule (summarized in [96]), no conclusive information has been provided for plague. Medical, anthropological and historical studies have inspected sex or gender differences during plague outbreaks of the past (reviewed in [42]) with contrasting results. Rarely was it possible to reconstruct mortality rates for pre-industrial plague epidemics: one exception is the valuable work of Alfani *et al*. [57] on the plague of Carmagnola (Italy, 1630), which was accomplished by cross-referencing several historical data to obtain a dataset with indication of sex, age and outcome, albeit without any indication of plague form.

To address the question as to whether to a high number of plague cases corresponds to a high number of deaths in one of the two biological sexes or at a certain age and to discuss the possible causes of the evidence, we collected modern medical plague datasets, which, in comparison to data from skeletons of plague victims and historical registers, provided us with additional information (besides sex and age) about plague form and outcome of the disease for people before the antibiotic era (1720–1945 CE). The data collected from different sources (electronic supplementary material, S1) concerned more than 1600 cases recorded worldwide. The goal of our work was to better understand whether biological or cultural reasons accounted for plague cases and deaths in the different macro-areas.

From an initial dataset, we excluded septicaemic and pneumonic plague and retained only those affected by bubonic plague whose sex, age and outcome were known (967 total cases).

**Figure 4.**
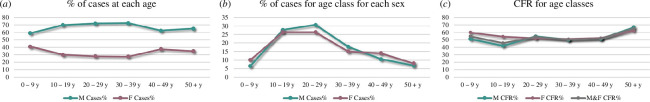
Trends of cases and CFR in both sexes at different ages. (*a*) Relative frequencies of male and female cases on the total of cases for each age class; (*b*) for each sex separately, frequencies of cases at each age class; and (*c*) CFR% of males and females at each age class.

A first interesting finding concerns the prevalence of male individuals infected by plague in general (figure 1) and bubonic plague in particular (figure 4*a*), worldwide: in our database, 70% of hospitalized cases with evident buboes were male (while 30% were female), with a male prevalence within each macro-area (table 1) and for each age (table 2, figure 4*a*), with no exception. A similar trend was observed in the second half of the twentieth century in Madagascar, with plague affecting more men (57.1%) than women (42.9%) in a dataset of 20 900 notified cases [97]. Thus, at least for pathogenicity, bubonic plague apparently mirrors the tendency of other epidemic diseases.

Cultural aspects like gender-specific occupational and social activities are considered as factors that increase the exposure to infections in the review of WHO about sex-specific epidemic-prone diseases [58]. In general (although with exceptions), men are more exposed to infection because they tend to be outside home longer than females. Another reason for a larger number of males in our medical reports may be owing to a form of social discrimination or inequity towards females, who might have had less access to resources (see also [55]) like hospitals and treatments [58]. Females are also more often carer, at both familial and social levels, a condition which could also prevent hospitalization [55,57]. Regarding plague, if cases are not rapidly isolated, human-to-human contagion owing to direct contact with sick bodies or with vectors like fleas or lice may occur, a condition that has been recently re-proposed (e.g. [9,56,98–100]), and women could be infected at the same rate as men, but they remain home.

The observed trend (more male cases worldwide, including Madagascar [96]) might rather suggest that biological factors make the male sex more susceptible to bubonic plague. In fact, gender-specific behaviours and/or gender inequalities are not the only mechanism proposed by the WHO to explain the increased risk of infection for males; biological factors play an equally important role, in general. There are at least two biological mechanisms, which are known to work for infectious diseases. (i) At the genetic level, on the X-chromosome, regions containing immune-related genes are present, which may clear infections at early stages. Two X-chromosomes may provide females with greater plasticity in their immune response against pathogens than males (reviewed in [101]; see also [96]). (ii) The second mechanism is mediated by sexual hormones: testosterone inactivates the immune system, while oestrogen acts as an activator [102,103]. In fact, multiple immune-related cells (including neutrophils [92]) are partially controlled by oestrogen, for which they express ER-α and ER-β receptors (reviewed in [101]). Female neutrophils appear more activated and mature than male ones, owing to hormonal regulation [104], and might therefore provide more defence in the early phases of infection, thus resulting in negative or not identified plague cases. In the case of bubonic plague, a third defence mechanism for females might be owing to reduced compartmental iron content, a circumstance that may make females from adolescence less likely to be infected by bacteria like *Y. pestis*, which require extracellular iron for their growth and multiplication [105,106]. Notably, the absence of the Yersiniabactin (Ybt) siderophore-dependent system or the Yfe iron transporter attenuate the virulence of *Y. pestis* in mouse models [107,108]. Hypoferremia in females has long been associated with the menses, yet recent studies [109,110] have demonstrated that oestrogen actively reduces the uptake of iron from the intestine by promoting the production of hepcidin, whereas testosterone inhibits its production. Thus, oestrogen reduces in females the quantity of circulating iron from adolescence through maturity until menopause, perhaps contributing to diminishing female bubonic plague cases.

Observing figure 4*a*, we can see that, while male frequencies of cases are prevalent on female incidences at each age, female relative frequency of cases decreases in the reproductive period (age classes from 10–19 to 30–39 years) when the effect of hormones and iron combined might account for less susceptibility in women. Yet, comparing the relative frequencies of cases at each age class in both sexes (figure 4*b*), we do not see any differences: the tendency of females exactly mirrors that of males and shows the same risk of susceptibility for both sexes at any age class, suggesting that in the case of bubonic plague infection, biological differences cannot be responsible for the immune response to the pathogen. More likely, exposure owing to occupational and social activities or gendered behaviours, thus cultural aspects globally makes both sexes more prone to be affected and hospitalized during the age of 10–39 years. Indeed, in Kenya, Mozambique and Tanzania, 1986–2002 [111,112], women and children were reported to be mostly affected by plague. It should be noted that these studies did not differentiate for the form of plague, although septicaemic and pneumonic have different mechanisms and different lethality (in our dataset, for instance, females were more affected than males by pneumonic plague; figure 1). Regardless, the authors attribute this tendency to the habit of women and children sleeping on the ground, thus more in contact with parasites, whereas men sleep in beds [111]. Other examples of contrasting evidence for sex bias in bubonic plague incidence have also been collected by Pollitzer [113], whose authoritative review, which is the most recent work globally considering gender/sex-specific differences for plague, concluded that all sex differences observed for plague cases in different geographical contexts may be attributable to dissimilarities in exposure, thus to occupational, social and other cultural causes. Another cultural reason, unbalanced access to hospitalization by gender, could explain the large difference observed between males and females in our dataset.

Further, the majority of infectious diseases, which show higher susceptibility in males, show a higher mortality risk for males as well (e.g. [114]). Conversely, from our complete dataset of bubonic plague cases, we observe that infected females seem to be more prone to die in comparison to males (CFR 55.6% against 51.2%, data obtained from the sum of all macro-areas; table 1). Although the difference between sexes is not statistically significant, this evidence confirms that females are not more protected than men by their immune system in the course of the disease. This phenomenon is particularly evident in Europe (both Second and Third Pandemics; table 1) and India, but the opposite can be observed in Australia and East Asia. In fact, we observe globally (figure 4*c*), that females show a maximal CFR during childhood and adolescence when the hormones have not yet reached their peaks (sexual hormones peak in the mid- to late-20s), as expected. Yet, adults do not show sex bias, although the hormonal levels, and thus the protecting effect in females, should be higher. In fact, in (female) individuals with less iron in the extracellular compartment, hepcidin causes retention of iron within the macrophages owing to clearance of red blood cells. Thus, if the infection is not early cleared by neutrophils, it could be that *Y. pestis* finds in iron-overloaded macrophages a much more suitable environment for its proliferation, thus might develop in a more aggressive form of bubonic plague in females, owing to a higher number of bacteria in the lymph nodes, from adolescence (thus justifying the notable, although not significant, difference in CFR between adolescent males and females). While remaining a hypothesis, the presence of iron overload in the macrophages, which is hepcidin-dependent, should be enhanced under the influence of oestrogen and reduced under the effect of testosterone (thus from the mid-20s years of age). These contrasting effects of the hormones might explain the similar trend observed in male and female CFRs in adulthood.

Another hypothesis is that women of fertile age were admitted to hospitals only if they were pregnant, a condition that might enhance the risk of complications. In our dataset, pregnancy was reported in 12 cases worldwide, albeit only five were considered in our final dataset, of whose both age and outcome were recorded. In fact, there is no reason to hide a pregnancy in a medical report; conversely, the case of a plague-infected pregnant woman is interesting. In a systematic review [115] conducted worldwide on pregnant women between the ages of 18 and 40 infected with plague, the authors found at least 59 eligible papers published from 1897 to 2002, with 160 cases reported in detail, with information on the course and outcome of disease for both mother and fetus/child, also in the pre-antibiotic era.

In figure 4*c*, we can also see that older people (50+) are mostly prone to die from bubonic plague, regardless of sex, possibly owing to the reduced presence of hormones and to iron-loss. Thus, it could be that mortality owing to bubonic plague is affected by sex- and age-related factors owing to contrasting biological effects, although experimental work is necessary to verify this hypothesis.

Further, we tried to apply statistical models to detect eventual predicting factors of death for bubonic plague. To the best of our knowledge, only two studies [57,97] used logistic regression analysis to infer risk factors of dying for plague. The first one [57] found a prevalence of female cases in historical data from Carmagnola (seventeenth century), whereas the other [97] confirmed the prevalence of male cases at almost each age class previously observed in Madagascar [116,117]. The first work [57] observed a slight disadvantage of males in surviving plague and in individuals at age 41–60, regardless of sex. In the other work [97], neither sex (M versus F 0.98 : 1.00 OR, with 95% CI), nor the adolescent age (children 0–9 versus adolescents 10–19, 1.00 : 0.88 OR and OR_a_, with 95% CI) were associated with an enhanced risk of dying for plague in Madagascar. Although bubonic plague was prevalent (92.6 %) in the dataset of Migliani *et al*. [97], no disaggregation for plague form was performed in their logistic regression analysis, whereas in the Alfani *et al.* [57] dataset, the form of plague was unknown. In addition, in the case of Madagascar, the 4309 cases under investigation, which showed similar mortality rates in males (24.2 %) and females (24.5 %), were collected from 1957 to 2001, and the patients were in part treated with different antibiotic therapies (for plague therapies, effectively started in 1947; see [41]).

In the present work, logistic regression analysis confirmed the evidence that being old enhances the risk to die of bubonic plague (*p*‐value = 0.088). Apparently, sex does not significantly affect the risk of dying, yet females have a slightly higher probability, which may confirm the previously proposed biological mechanism. Regardless, the only significant risk factors evinced was the geographic macro-areas of infection and treatment, with a significantly higher risk to die from bubonic plague in India and East Asia and a significantly lower one in Australia, in comparison to Europe (Second and Third Pandemics). (We also tested the European Second and Third Pandemics separately, without observing any significant change—electronic supplementary material, table S5.) We cannot exclude that regional dissimilarities are owing to population genetic differences (this point would also deserve further investigation in light of new evidence [118]), but we should consider that the Australian population has a very recent European ancestry (end of the eighteenth century), which cannot explain the discordant results between the two macro-areas. Possibly, there are different cultural behaviours in distinct parts of the world that may influence the risk to die from plague. For instance, women might have been admitted to hospital only at a late stage of the disease, a condition which may have enhanced their probability of dying of bubonic plague and affected our observation of a higher risk for females. However, the benefit of being hospitalized at an earlier stage of bubonic plague in the pre-antibiotic era may only relate to better access to food and water (as proposed by [57]), since we found no evidence of antimicrobial therapies in the reports. Only in the late 1940s was carbolic acid experimentally employed in Hong Kong, although with mixed results [73] (electronic supplementary material, S1). Both in Australia and Europe (Third Pandemic) serum was used, albeit with discordant results: of the 341 total cases in Australia, 248 were treated with serum (Yersin-Roux serum produced by Pasteur Institute in Paris from 1901 to 1904; from 1904 to 1907, the same serum employed was produced by the Lister Institute of Preventive Medicine in London [84]). Table 4 showcases that the use of serum halved the general risk to die for bubonic plague in Australia (total CFR 25% against 51.6%, table 4), working more efficiently in females than in males (CFR 17.2% against 26.8%)—albeit the natural CFR is as well lower in females than in males in Australia (CFR 45.8% against 53.6%). In Europe, the Pasteur serum was employed at least during the outbreak in Taranto 1945 (Italy) [72] along with an antimicrobic therapy on 17 individuals, of whom only 7 survived. To our knowledge, the serum was not employed in the other European countries represented in our dataset.

**Table 4.** Bubonic plague patients treated or not with Yersin–Roux serum during the Third Plague Pandemic in Australia. The prevalent CFRs are reported in bold.

	M			F			M&F		
	cases	deaths	CFR%	cases	deaths	CFR%	cases	deaths	CFR%
serum	190	51	**26.8**	64	11	17.2	248	62	**25.0**
no serum	69	37	**53.6**	24	11	45.8	93	48	**51.6**
total	259	88	**34.0**	86	22	25.6	341	110	32.2

Besides treating plague patients with serum, Australia also applied safety measures from the beginning of the plague in Hong Kong in 1894 to prevent the entry of plague through its ports, thereby significantly reducing the number of cases [85]. Government and public health authorities were able to stop the various outbreaks that struck Australia from 1900 to 1925. Similarly, previous work on data from the Third Plague Pandemic in Europe [9,119] had proposed that prevention and control measures, thus cultural behaviours, led to a reduced number of cases and to the end of plague in Europe.

In conclusion, and apparently, cultural differences as well as biological reasons may account for dissimilarities in infection and mortality risk owing to bubonic plague. Although further studies are needed to verify our hypotheses, it is clear that a gender/sex perspective might be of great help in better understanding cultural and biological phenomena behind infectious diseases. With WHO [58], we strongly recommend that data should always be accomplished separately by sex and along with information on age, disease outcome, occupational activity and other elements, which can help to better understand differences in disease course and outbreak evolution.

## 4. Material and methods

### 4.1. Material

The database used in this analysis includes individual data of 1616 cases of plague victims and their course. The data include cases of epidemics in several countries of the world from 1720 to 1945 (figure 1). Information was drawn from 33 publications (electronic supplementary material, table S1).

### 4.2. Data collection

We systematically searched for online publications (using online databases and collections such as Google Scholar, Internet Archive, Wellcome Collection, Google Books) that provided detailed information on the infection and course of plague patients. For our research, we used the following keywords: ‘plague’, ‘third plague pandemic in Europe’, ‘plague in’, ‘medical reports of plague’, ‘clinical data of plague victims’, ‘cases of plague’ in combination with ‘recovery’, ‘healed’, ‘sex’, ‘age’, ‘death’. We extended the research also to publications in different languages, like Italian, French and Spanish. We included all publications in which individual data of plague patients, such as age and sex, were reported and excluded all cases with an uncertain diagnosis of plague and whose outcome of the infection was not known. The 33 publications selected produced data on 1616 individuals from 17 different countries. For this study, we further selected our data to include only individuals infected by bubonic plague (i.e. all cases in which the presence of the bubo was specified), of known sex and age and whose disease course (death or recovery from plague) was indicated, for a total of 1154 cases.

### 4.3. Statistical analysis

Descriptive statistical analyses (means, standard deviations and frequencies) were performed for all the study variables. The CFR (i.e. percentage of individuals who died divided by diagnosed cases of plague) was calculated for each sex and age classes. Comparisons between categorical data were performed using chi-square test. Multivariate logistic regression model (logit model) analysis was performed to test the association between biological features (sex and age) and geographic macro-areas as categorical predictor variables and death by plague/recovering from plague being the binary outcome. Sex and age were included as categorical variables and age was divided into three age classes: juveniles (0–14 years), adults (15–49 years), and older adults (more than 50 years). Geographic macro-areas considered are Europe (Second and Third Pandemics), East Asia, Australia and India. Male category, middle age class (adults) and Europe were used as reference values. The results were reported as ORs with 95% CI. All the assumptions of the logistic regression were met (a binary dependent variable; no multicollinearity among the independent variables; independence of the observations; the sample size respects the ‘rule of thumb’). The significance of each regressor is assessed by the Wald test and its *p*‐value. Cox and Snell *R*^2^ and Nagelkerke *R*^2^ were reported. The validation of the model was performed using the Hosmer–Lemeshow goodness-of-fit test. Participants with missing data were excluded from the analysis.

Values of *p* < 0.10 were considered statistically significant [48,57]. All statistical analyses were conducted using STATISTICA (version 11, StatSoft, Tulsa, OK).

## Data Availability

Data are reported in the electronic supplementary material [120].
